# Dual-room twin-CT scanner in multiple trauma care: first results after implementation in a level one trauma centre

**DOI:** 10.1007/s00068-020-01374-5

**Published:** 2020-04-25

**Authors:** Maximilian Kippnich, Nora Schorscher, Markus Kredel, Christian Markus, Lars Eden, Tobias Gassenmaier, Johann Lock, Thomas Wurmb

**Affiliations:** 1 Department of Anesthesia and Critical Care, Subsection Emergency- and Disaster Relief Medicine, University Hospital of Wuerzburg, Oberduerrbacherstrasse 6, 97080 Würzburg, Germany; 2 Department of Anesthesia and Critical Care, University Hospital of Wuerzburg, Würzburg, Germany; 3 Department of Trauma Surgery, University Hospital of Wuerzburg, Würzburg, Germany; 4 Department of Diagnostic and Interventional Radiology, University Hospital of Wuerzburg, Würzburg, Germany; 5 Department of General and Visceral Surgery, University Hospital of Wuerzburg, Würzburg, Germany

**Keywords:** Trauma centre, Trauma management, Resuscitation time, Dual-room whole-body CT

## Abstract

**Purpose:**

The trauma centre of the Wuerzburg University Hospital has integrated a pioneering dual-room twin-CT scanner in a multiple trauma pathway. For concurrent treatment of two trauma patients, two carbon CT examination and intervention tables are positioned head to head with one sliding CT-Gantry in the middle. The focus of this study is the process of trauma care with the time to CT (tCT) and the time to operation (tOR) as quality indicator.

**Methods:**

All patients with suspected multiple trauma, who required emergency surgery and who were initially diagnosed by the CT trauma protocol between 05/2018 and 12/2018 were included. Data relating to time spans (tCT and tOR), severity of injury and outcome was obtained.

**Results:**

110 of the 589 screened trauma patients had surgery immediately after finishing primary assessment in the ER. The ISS was 17 (9–34) (median and interquartile range, IQR). tCT was 15 (11–19) minutes (median and IQR) and tOR was 96.5 (75–119) minutes (median and IQR). In the first 30 days, seven patients died (6.4%) including two within the first 24 h (2%). There were two ICU days (1–6) (median and IQR) and one (0–1) (median and IQR) ventilator day.

**Conclusion:**

The twin-CT technology is a fascinating tool to organize high-quality trauma care for two multiple trauma patients simultaneously.

## Introduction

The major reason for fatal outcome in trauma patients is the delay in life-saving surgery [1]. In general, the indication for surgery is set by clinical and radiological diagnosis. The diagnostic work-up for patients with multiple trauma has undergone a paradigm shift from the conventional approach (ultrasound and conventional radiography) to whole-body CT as the first diagnostic tool used in trauma centres [2–7]. For this purpose, a CT scanner is located directly in or nearby the resuscitation area of the emergency room or trauma suite. This has resulted in faster diagnostics and treatment intervals [8–10]. CT is widely accepted as the gold-standard early diagnostic tool in patients with multiple injuries. If performed quickly, it is feasible even in haemodynamically unstable and severely injured patients [11]. The direct effect of this approach on the mortality and morbidity of trauma patients is still controversial and fiercely debated [12–14].

Since 2004, our level one trauma centre successfully uses whole-body CT as the first-line diagnostic tool in trauma patients with multiple injuries. Over the years, we have optimized the treatment process by defining standard operating procedures. After 14 years of clinical and scientific experience with this concept, we redefined the concept in 2018. This became necessary to fulfil the needs of growing numbers of trauma patients and to use the benefits of the newest technical scanner capabilities.

**Fig. 1:**
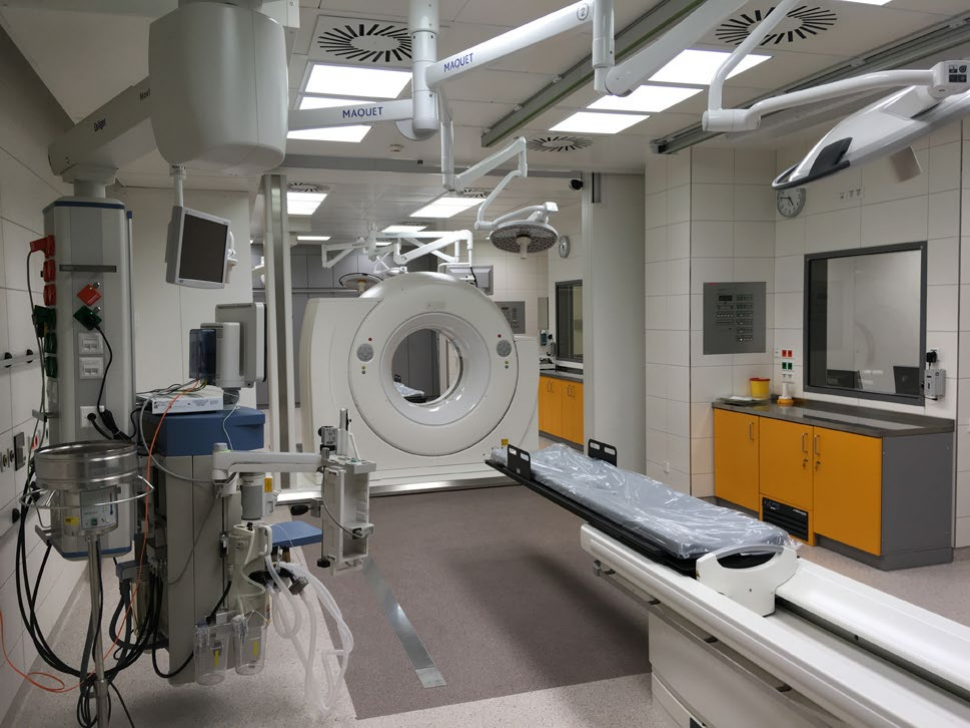
Large-scale trauma resuscitation area with a dual-room sliding gantry CT scanner

The new trauma resuscitation room is configured as “two rooms in one”. For the concurrent treatment of two trauma patients, two carbon CT examination and intervention tables are positioned head to head with one sliding gantry CT in the middle (Fig. 1). Important devices, such as ventilator, ventilator tubing and central venous lines remain unaffected in position during the whole-body CT scan. In order to protect the staff and the second patient from radiation, a mobile wall can separate both resuscitation rooms.

The main aim of the study is to evaluate the process of trauma care based on a dual-room whole-body CT. Therefore, we analyzed different sub-steps of the overall process in those patients who required emergency surgery. The time intervals from the arrival of a patient in the resuscitation room to the start of CT and of emergency surgery has been defined as the main quality indicator for the whole process of trauma resuscitation.

Moreover, we intended to identify possible problems and pitfalls of this new concept and started the data collection directly after introduction of the concept without having had any training or familiarization period. This allowed for a systematic evaluation and eventually necessary trouble shooting after the first 6 months of installation.

## Methods

### Protocol design

This investigation is a retrospective data analysis based on a chart review. The project was presented to the local ethics committee of the University of Wuerzburg and was exempted from the need of ethical approval (Reference number 2019013001).

We screened all trauma patients that were treated in the trauma resuscitation room during the first 6 months after introduction. We identified those patients, who required emergency surgery, anonymized the data and entered it into an abstract form. For those patients, we collected the time to CT (tCT), the time to emergency surgery (tOR), the injury severity score (ISS) and outcome variables (mortality, ventilator-dependent days and length of intensive care unit (ICU) stay). These variables are routinely recorded in the hospital’s clinical information system. The data were analyzed retrospectively.

### Setting

The Wuerzburg University Hospital is a certified Level One Trauma Centre. In 2018 it underwent reconstruction as described above. A new and large-scale trauma resuscitation area with a dual-room sliding gantry CT scanner for simultaneous treatment or two multiple trauma patients by its was introduced (SOMATOM Definition Edge, Siemens Healthineers, Erlangen, Germany).

The trauma algorithm of our centre was established in 2004 and was adjusted to the new circumstances in 2018. It is described below:

In our institution, leadership of the trauma team is an interdisciplinary approach with a ‘leading group’ consisting of the senior anaesthesiologist, the senior surgeon and the senior radiologist. The trauma team is always consists of:

- Two anaesthesiologists (senior and resident);
- Two surgeons (senior and resident);
- One trauma surgeon (senior or resident);
- One radiologist (senior);
- Two anaesthetic nurses, one to two surgical nurses and two radiology technicians.

Neurosurgery, heart and thoracic surgery, urology, paediatric surgery, maxillofacial surgery and ENT specialist consultants are available within 15 min twenty-four-seven.

If two patients are announced at the same time, the trauma team will be expanded accordingly.

After handover of the patient by the emergency medical service, the patient is transferred from the ambulance stretcher directly onto the examining table of the CT scanner. Any life-saving procedures, including airway management, emergency laparotomy or thoracotomy can be performed on this carbon CT examining and intervention table. Before starting the CT, a physical exam is performed according to the priority-based standard of care and life-threatening conditions are treated immediately according to ATLS^®^ and the Department’s Standard Operating Procedures. Focused assessment with sonography for trauma (FAST) is performed only on haemodynamically unstable patients or if the ‘leading group’ decides to do so. If the triage rule for whole-body CT applies to the major trauma victim, the CT scan starts after resolving life-threatening problems [6].

After the scans have been completed, the algorithm continues with a reassessment followed by further stabilization and planning the next steps, e.g. transport to the operating room (OR) or to an ICU.

### Inclusion criteria

Patients with suspected multiple trauma, who required emergency surgery who were initially diagnosed by the CT trauma protocol between 05/2018 and 12/2018. Emergency surgery was classified as any kind of surgery that had to be performed immediately after finishing the resuscitation period and the diagnostic work-up.

### Exclusion criteria

Patients who required immediate emergency laparotomy or thoracotomy directly in the trauma resuscitation room; patients who were not transferred directly to the patients without getting a complete diagnostic work-up.

### Methods and measurement

Time to CT (tCT) and time to emergency surgery (tOR) as two important quality indicators (process) in trauma emergency care were analyzed. These were defined as the time interval from patient’s arrival in the emergency room until the start of the CT respectively of the emergency surgery in the operating theatre. This included the time needed for all life-saving and diagnostic procedures, transfer, positioning and preparation for CT or for surgery. For detailed analysis, the patients were divided into three different subgroups according to their ISS (minor injury ISS 0–15, moderate injury ISS 15–24, severe injury 25–75). The calculation of AIS and ISS was performed by one investigator, trained in these methods. A supervisor was in charge in unclear cases. Outcome variables (mortality, ventilator—dependent days and length of ICU stay) were determined and are listed purely descriptively. Owing to various influencing factors, these variables can only give information of possible tendencies. Trauma mechanism and the different surgical procedures were recorded.

### Historical control group

The historical control group includes all patients with suspected multiple trauma, who required emergency surgery and who were initially diagnosed by using the CT trauma protocol between 2004 and 2006. In this time, the trauma resuscitation room was configured with a single-room whole-body CT. There was no simultaneous treatment.

### Analytical methods

There was no normal distribution in the Kolmogorov–Smirnov test. The data are shown as the median with inter-quartile range (IQR 25–75% percentile). The non-parametric Mann–Whitney rank sum test was used. Statistical analyses were performed by IBM SPSS and Microsoft Excel software and *p* < 0.05 was considered significant.

## Results

In the study period (05/2018–12/2018), a total of 589 trauma patients were screened. 110 of all screened patients had surgery immediately after finishing primary assessment in the ER. Those were included in the analysis.

42 patients were excluded (20 without a complete diagnostic work-up, 3 of them had emergency laparotomy or thoracotomy direct in the trauma bay; 22 not transferred directly to the OR).

224 of the 589 screened trauma patients were treated simultaneously with the dual-room whole-body CT. That means, that in 112 cases two patients were at the same time in the trauma resuscitation room. 12 of the 224 patients had immediate surgery simultaneously. Owing to the retrospective design of the study, more detailed analysis of this subgroup was not possible.

### Surgical procedures and mechanism of trauma

Trauma mechanism and the different emergency surgical procedures are shown in Table 1. All limb fractures were open and/or dislocated fractures. The indication for urgent and immediate surgery was therefore set by the trauma surgeon. Abbreviated injury scale for fractures was 3 or greater. All patients with spine surgery had instable vertebral fractures.

**Table 1:** Surgical procedures and mechanisms of trauma

Surgical procedures	
Craniectomy	14
Laparotomy	17
Upper limb	16
Lower limb	41
Thoracotomy	9
Spine	5
Pelvis	8
Mechanism of trauma	
Car accident	32
Motorbike	24
Pedestrian	4
Fall > 3 m	18
Others	32

### ISS and descriptive outcome parameters

The median ISS was 17 (9–34) (median and IQR). In the first 30 days, seven patients died (6.4%) including two within the first 24 h (2%). There were two ICU days (1–6) (median and IQR) and one (0–1) (median and IQR) ventilator day.

### tCT and tOR

**Fig. 2:**
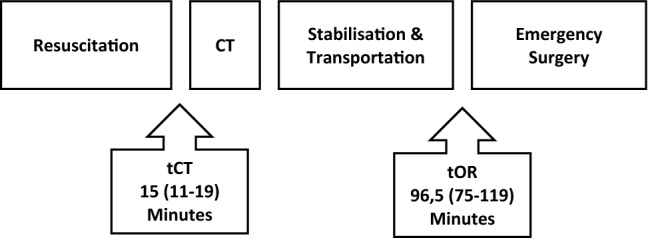
Process of trauma care with the quality indicators time to CT (tCT) and time to operation (tOR)

tCT was 15 (11–19) minutes (median and IQR) and tOR was 96.5 (75–119) minutes (median and IQR) (Fig. 2). Urgent and emergency laparotomies were performed after 85 (80––96) minutes (median and IQR), minimum after 36 min, urgent and emergency craniectomies after 93 (60–138) minutes (median and IQR), minimum after 43 min, after arrival in the hospital (ISS > 24).

### Comparison to a historical control group with a single-room whole-body CT

Table 2 shows detailed results of the tOR and the descriptive outcome parameters in the three subgroups (ISS 0-15, ISS 16-24 and ISS 25-75) compared with a historical control group.

**Table 2:** Detailed results of the subgroups ISS 0–15, ISS 16–24, ISS 25–75

ISS 0–15	Group 1 (*n* = 50)	Group 2 (*n* = 21)	*p* value
tOR	103 (79–118)	100 (95–125)	0.266
ISS	9 (9–10)	12 (10–13)	< 0.05*
Mortality (24 h)	None	None	
Mortality (30 days)	None	None	
ICU days	1 (0–1)	0 (0–2)	0.541
Ventilator days	0 (0–1)	0 (0–2)	0.451

ISS 16–24	Group 1 (*n* = 17)	Group 2 (*n* = 48)	*p* value
tOR	102 (64–114)	110 (84–136)	0.089
ISS	19 (17–22)	22 (17–22)	0.454
Mortality (24 h)	None	None	
Mortality (30d)	1	None	
ICU days	2 (1–7)	3 (2–8)	0.635
Ventilator days	1 (0–1)	1 (1–3)	0.094

ISS 25–75	Group 1 (*n* = 43)	Group 2 (*n* = 94)	*p* value
tOR	90 (71–125)	105 (85–129)	< 0.05*
ISS	43 (31–52)	41 (29–48)	0.751
Mortality (24 h)	2	5	
Mortality (30d)	4	9	
ICU days	5 (3–17)	14 (7–23)	< 0.05*
Ventilator days	1 (1–6)	12 (5–21)	< 0.05*

Group 1 = Dual-room whole-body CT; Group 2 = Single-room whole-body CT

ISS, ICU days and ventilator days are shown as median and IQR, date for tOR are shown in minutes with median and IQR

*ISS*, injury severity score, *tOR* time to operation, *ICU* intensive care unit

*Statistically significant (*p* < 0.05)

## Discussion

Whole-body CT during the initial diagnostic work-up of patients with multiple trauma is mainly accepted as standard in trauma resuscitation care [7, 15]. Over the last years, there is growing evidence for its positive effects on outcome and mortality [9, 12–17]. After 14 years of positive experience with a sliding gantry-based “CT-First- Concept”, we constructed a new trauma resuscitation room based on a dual-room twin-CT system.

The presented study focused on the performance of the multidisciplinary team right at the beginning after introduction of our new trauma resuscitation room. In order to describe the performance of trauma care, we have used accepted quality indicators [15]. For our purpose, we focused in particular on the tCT and the tOR. The outcome variables (mortality, ventilator-dependent days and length of ICU stay) were used descriptively.

In our data, we found tCT a little longer when compared with the previous studies in our level 1 trauma centre (15 min vs. 10 min) [3]. In the previous studies, the trauma resuscitation room was based on a single-room whole-body CT with no simultaneous treatment. The longer tCT can be explained by the lack of excessive training of the multidisciplinary team with the new area. For example, the radiology technicians were trained, but unfamiliar to the preparation and patient positioning in order to correctly run the new dual-room sliding CT scanner system. The goal in the future is to optimize the resuscitation period and to start imaging after 10 min.

The tOR was reduced as compared with the former concept (historical control group); particularly, in the subgroup ISS 25–75 (90 min vs. 105 min) [12]. This may partly be attributed to the higher computing capacity and faster reconstruction capability of the CT scanner system.

Currently, there is no published data that focuses on the tCT and the tOR in a trauma resuscitation room with a dual-room sliding gantry CT system. Nevertheless, Weninger et al. described tOR for patients with blunt major trauma using a multi-slice computed tomography protocol with a single CT scanner. Their findings are comparable to our results as emergency surgery was started after 103 min in the MSCT cohort [18]. The results of the study show a significant difference in the tOR in favour of using the CT trauma concept. We observed similar results in our study. Especially in the subgroup of patients with severe injuries (ISS 25–75), we found a positive effect on time when compared with the former concept. The distribution of required surgical procedures and trauma mechanisms were comparable.

Some studies describe the treatment process after introducing dual-room CT concepts.

Wada et al. described the installation of a dual-room “IVR-CT system” (CT scanner system with interventional radiology features) in an emergency room of a level 1 trauma centre. CT examination, damage control surgery and transcatheter arterial embolization can be performed in this setting. He concluded that this system can improve the survival of severely injured patients [19]. In contrast, our trauma care focuses on a fast CT examination and an immediate transport to the operation theatre if indicated. Major surgery is very rarely performed in the trauma resuscitation room.

The Amsterdam Trauma Workflow, which is published by Fun Kon Jin et al., is based on a sliding gantry CT scanner in the trauma resuscitation room [8]. In a simulation study with predefined scenarios, they showed that a sliding gantry CT scanner serving two mirrored (trauma) rooms could provide early CT scanning in trauma patients without impacting regular and urgent CT scanning in the daily business [20]. This concept is comparable to our approach.

Frellesen et al. compared trauma workup times of a new dual-room sliding gantry CT with a former stationary single-room CT. During the single-room CT trauma room workflow, patients were transported to a separated CT room, whereas in the new trauma room workflow the patient remained on the CT table for scanning in resuscitation area. The median time from patient arrival in the trauma room until beginning of CT scan was significantly shorter in the sliding gantry CT group [21]. As in our study, the tCT was 15 min, tOR data were not reported by Frellesen et al.

Based on our data, we identified specific lessons learned and plan to improve medical, organizational and technical workflows in order to achieve an even shorter tCT and tOR in the future. Main identified points were team performance, reduction in unnecessary medical interventions before starting CT-scan and performing technical CT-preparation and medical interventions at the same time. This quality improvement process is currently in progress and will be finished after a couple of measures and consequences. The results of this process will be reported separately. Bearing in mind that we found reduction in tOR despite the limitations of working within a new environment and with new technology and workflows we assume, that with training, experience and adaptation to the growing dimension and complexity a better performance can be achieved within a few months. A further study will be necessary at that point in order to reflect these improvements and lessons learned.

The limitations of this study should be acknowledged. Our investigation is a retrospective study based on a chart review with a limited number of patients. In addition, the reported outcome data are mainly descriptive. The main limitation of the study is the lack of a comparison with two polytrauma patients in a single-room whole-body CT trauma resuscitation room. To better describe a possible effect of the new method, we have calculated the statistical analysis with a historical control group of our trauma center in a single-room whole-body CT trauma resuscitation room. To show a possible difference in time and outcome in patients treated simultaneously, we are actually planning a prospective follow-up study.

## Conclusion

From our experience, the twin-CT technology is a fascinating tool to organize high-quality trauma care for two multiple trauma patients simultaneously. With regard to the treatment process, we identified the need to improve the treatment process especially after finishing the CT procedure. This quality improvement process is currently in progress. With regard to outcome, further studies will be necessary to proof the advantage of a twin-CT technology in trauma care.
